# Minimally Invasive Approach Using 2 Posteromedial Portals for Preserving Ligament Integrity in Arthroscopic Pull-Out Technique for Posterior Cruciate Ligament Tibial Avulsion Fractures

**DOI:** 10.1016/j.eats.2025.103663

**Published:** 2025-06-13

**Authors:** Ryo Sasaki, Kazuya Kaneda, Kosuke Saito, Yuri Hiraishi, Taichi Nishimura, Teppei Hayashi, Masaki Nagashima, Hideo Morioka

**Affiliations:** a Department of Orthopaedic Surgery, NHO, Tokyo Medical Center, Tokyo, Japan; b Department of Orthopaedic Surgery, Keio University School of Medicine, Tokyo, Japan; c Department of Orthopaedic Surgery, International University of Health and Welfare Mita Hospital, Tokyo, Japan

## Abstract

Posterior cruciate ligament (PCL) tibial avulsion fractures, although rare, can lead to significant complications, such as nonunion, restricted range of motion, and knee instability, if not treated effectively. This report presents an arthroscopic pull-out technique using 2 posteromedial portals, allowing exclusive execution of the pull-out procedure from these portals. This approach minimizes damage to the anterior cruciate ligament and PCL, which are often compromised by traditional methods that use anterior portals requiring dissection between these ligaments. By avoiding disruption of the ligamentous anatomy, this technique not only enhances surgical safety but also optimizes the potential for achieving appropriate tensioning during repair. This article aimed to demonstrate the benefits of this technique in reducing PCL avulsion fractures while preserving ligament integrity and minimizing invasive measures.

If a posterior cruciate ligament (PCL) tibial avulsion fracture is not treated properly, it can lead to complications such as nonunion, limited range of motion (ROM), and instability of the knee.^1^ Radiographs, computed tomography (CT), and magnetic resonance imaging usually reveal an avulsed bone fragment. Conservative treatment with casting often is effective for undisplaced fracture types. However, partially and completely displaced fracture types generally require surgical intervention.^1^^,^^2^ Although traditional open reduction and internal fixation (ORIF) techniques have been used for displaced PCL avulsion fractures,^3^, ^4^, ^5^ advancements in arthroscopic techniques have led to an increasing preference for minimally invasive approaches. A systematic review demonstrated that arthroscopic surgery yielded significantly greater subjective and objective knee outcome scores than ORIF.^6^

The currently used arthroscopic pull-out technique that requires anterior portals and a posteromedial portal is considered minimally invasive compared with traditional ORIF.^7^ However, it still requires clearing the space between the anterior cruciate ligament (ACL) and PCL and inserting an arthroscopic camera, shaver, drill guide, or other instruments, raising concerns about potential damage to these ligaments. In addition, the surgical field obtained from the anterior portal may be insufficient to confirm the PCL attachment site, especially distally. This Technical Note describes a method that minimizes invasion of the ACL and PCL by creating 2 posteromedial portals and performing the entire pull-out technique through these 2 portals, thus eliminating the need to clear the space between the ACL and PCL (Video 1). Written informed consent was obtained from the patient for the publication of this technical note and accompanying images. The datasets used in this study are available from the corresponding author upon request.

## Surgical Technique

### Preparation

**Fig 1:**
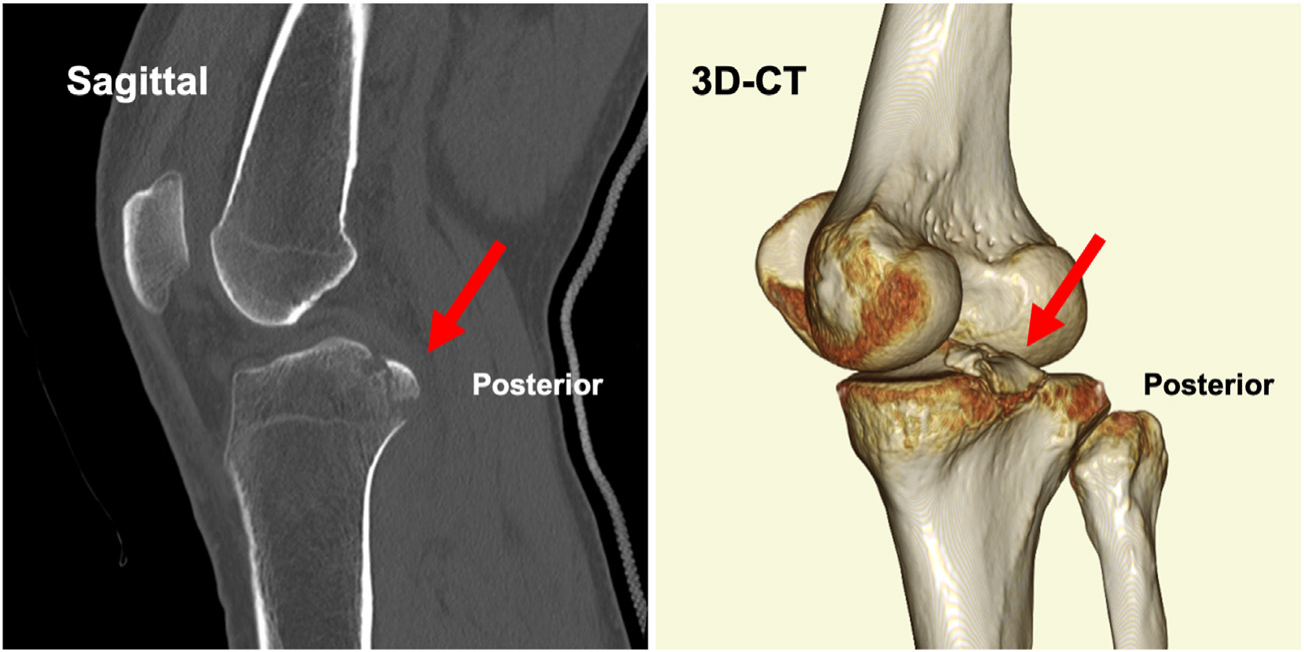
Preoperative sagittal and 3-dimensional computed tomography (3D-CT) in the right knee. A tibial avulsion fracture of the posterior cruciate ligament is observed. The main bone fragment is hinged type (arrow).

**Fig 2:**
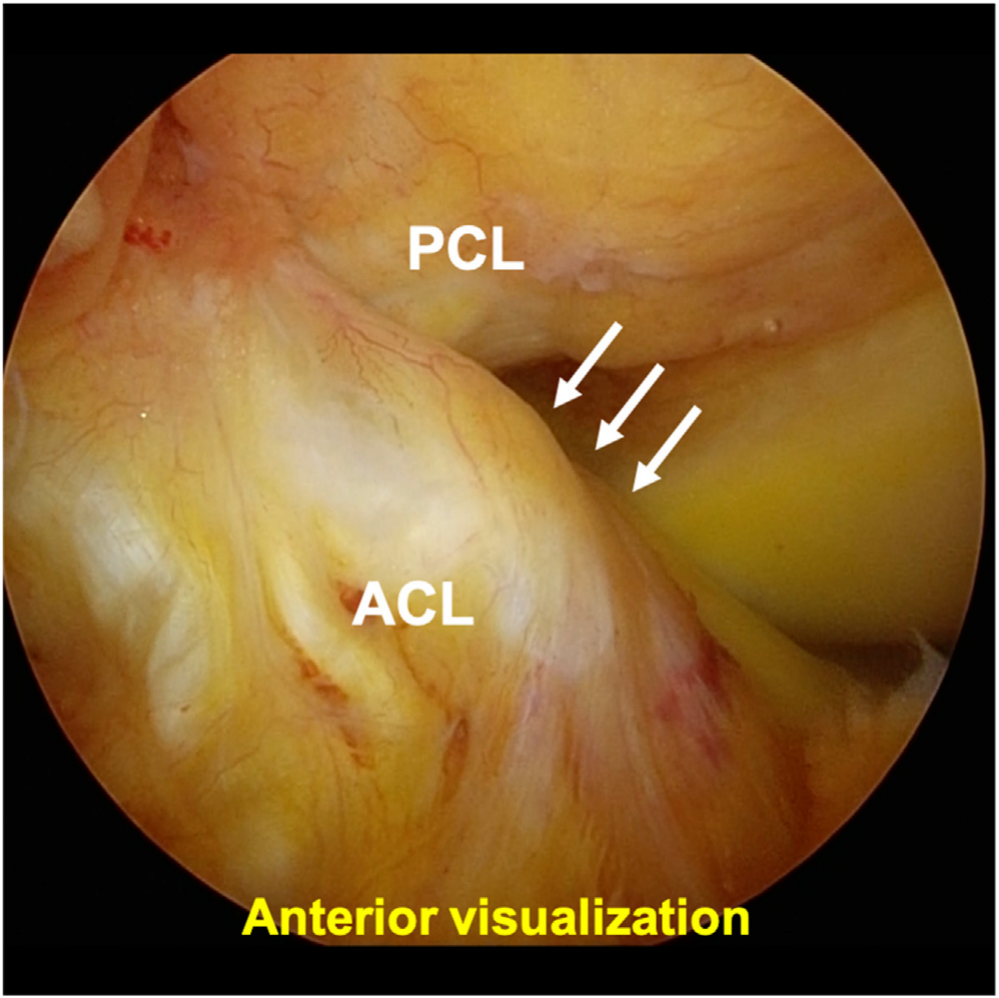
Preoperative anterior visualization of the arthroscope in the right knee from the anterolateral portal, positioned supine with the knee flexed at 90°. In the standard anterior visualization, the anterior cruciate ligament (ACL) is lax (arrows) owing to sagging caused by posterior cruciate ligament (PCL) malfunction.

The steps of the surgical procedure are listed in Table 1. For preoperative preparation, CT is performed to evaluate the displaced tibial eminence avulsion fracture (Fig 1). Under general anesthesia, the patient is placed in a supine position on an operating table with a standard leg holder, allowing full ROM. Standard knee arthroscopy is performed through the anterolateral and anteromedial portals. Complete diagnostic arthroscopy is performed initially. The PCL is probed to ensure that the femoral attachment is intact and there are no intraligamentous tears. In this case, the ACL is lax as the result of sagging caused by PCL malfunction (Fig 2).

**Table 1:** Procedure Tips and Tricks On the Basis of Our Experience

**Fig 3:**
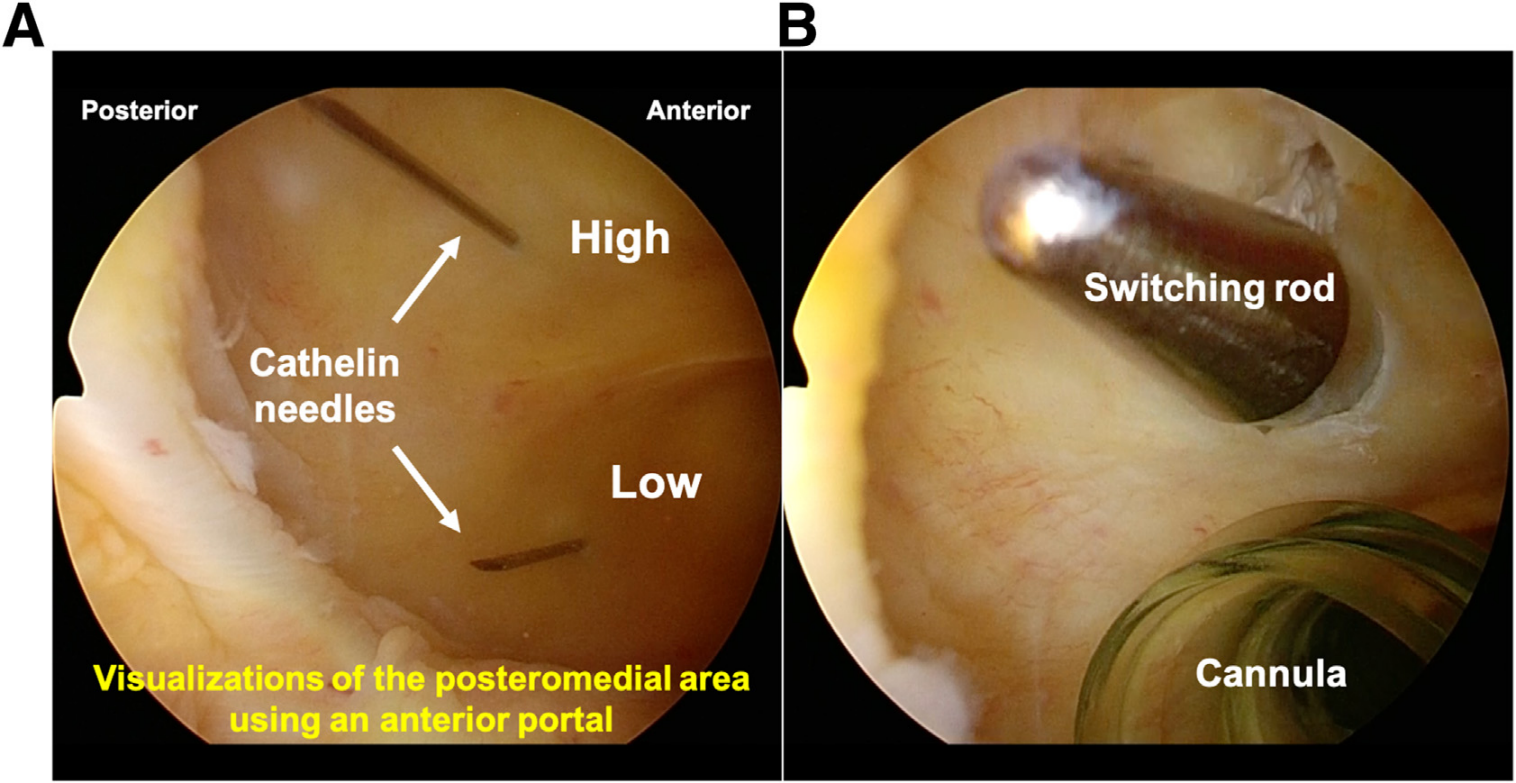
Creating 2 posteromedial portals in the right knee, positioned supine with the knee flexed at 90°, viewed from the anteromedial portal. Visualizing the posteromedial area using an anterior portal, two Cathelin needles are inserted to create high and low posteromedial portals (arrows) (A). Using the Cathelin needles as a guide, incisions are made and a cannula is inserted into the low posteromedial portal. The arthroscope is introduced through the high posteromedial portal using a switching rod (B).

**Fig 4:**
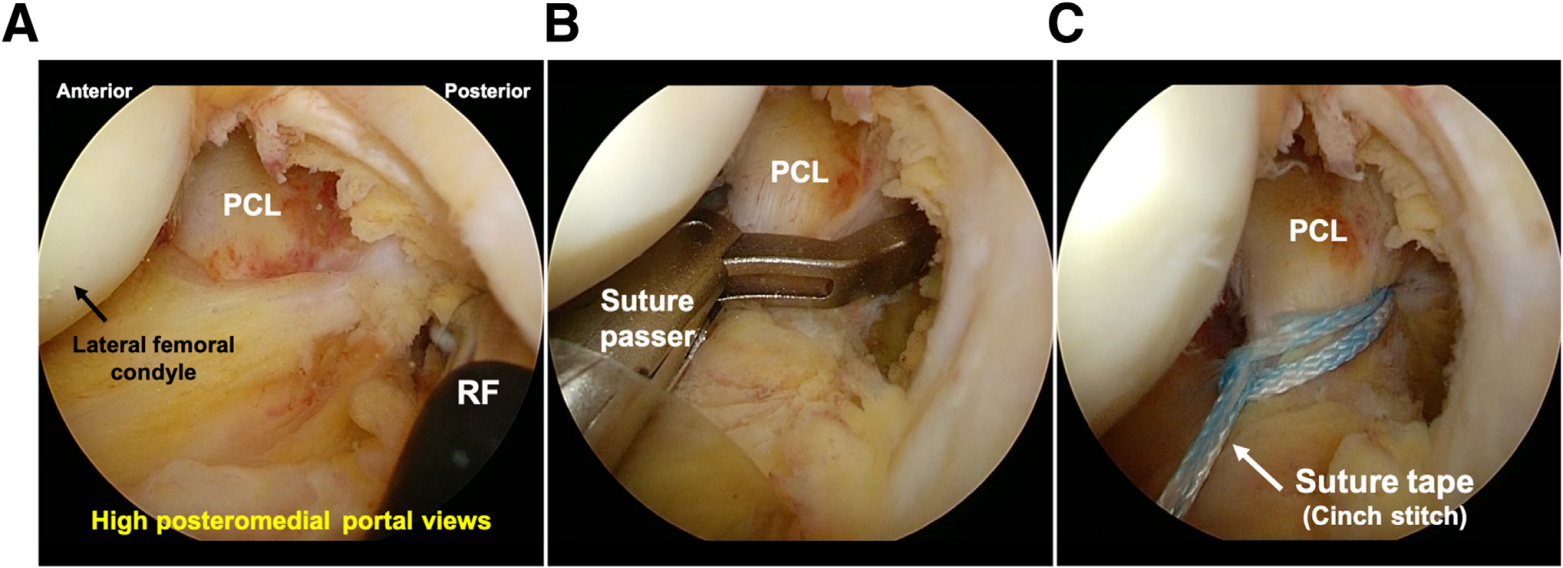
Suturing of posterior cruciate ligament (PCL) from the low posteromedial portal in the right knee, positioned supine with the knee flexed at 90°, viewed from the high posteromedial portal. After clearing the tibial bone tunnel creation area using a shaver or radiofrequency system (RF) from the low posteromedial portal in high posteromedial visualization (A), suture tape (ULTRATAPE; Smith & Nephew Endoscopy) is sutured to the PCL using a cinch stitch with a suture passer (FIRSTPASS MINI; Smith & Nephew Endoscopy) from the low posteromedial portal (B and C).

**Fig 5:**
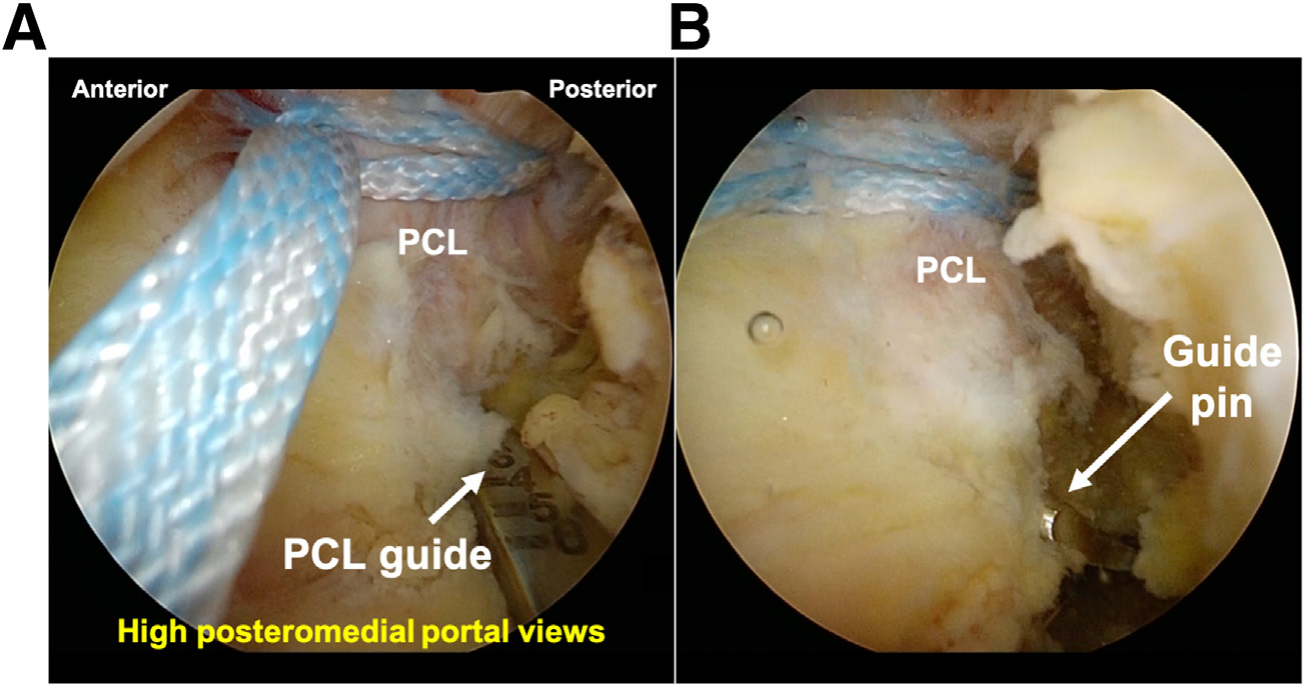
Inserting the guide pin to the fracture site in the right knee, positioned supine with the knee flexed at 90°, viewed from the high posteromedial portal.A posterior cruciate ligament (PCL) guide (white arrow) is then inserted from the low posteromedial portal (A), and a guide pin (Smith & Nephew Endoscopy) (white arrow) is placed through a 2-cm skin incision on the medial side of the tibial tuberosity to the center of the main bone fragment (B).

Indications • All PCL tibial avulsion fractures that can be repaired using an arthroscope.
Preparation • Perform standard knee arthroscopy using anterolateral and anteromedial portals (Fig 2).
Create 2 posteromedial portals • Visualize the posteromedial area from the anteromedial portal. • Insert 2 Cathelin needles to create high and low posteromedial portals (Fig 3A). • Insert a cannula or a slider for a meniscus suturing device to the low posteromedial portal. • Insert the arthroscope through the high posteromedial portal using a switching rod (Fig 3B).
Pull-out technique from only posteromedial portals • Clear the tibial bone tunnel creation area using a shaver or radiofrequency system from the low posteromedial portal (Fig 4A). (If there is considerable displacement of the fracture, remove debris from the fracture site.) • Suture the suture tape to the PCL using a cinch stitch with suture passer from the low posteromedial portal (Fig 4 B and C). • Insert a guide from the low posteromedial portal and insert a guide pin from a 2-cm skin incision on the medial side of the tibial tuberosity (Fig 5 A and B). • Confirm the position of the guide pin using C-arm imaging and drill with an EndoButton drill (Smith & Nephew Endoscopy) (Fig 7). • Insert the loop of the ETHIBOND suture through the EndoButton drill from the anterior tibial surface using a suture retriever, then retrieve it through the low posteromedial portal with a suture retriever (Fig 8A). • Relay the suture tape using the loop of the ETHIBOND suture (Fig 8 B and C).
Tensioning and fixation • Install the TensionLoc (Arthrex) in the anterior tibial bone tunnel and temporarily fix the suture tape. • Adjust to the appropriate tension while confirming that the bone fragment is not floating through C-arm imaging, that the PCL is properly tensioned under arthroscopic anterior visualization, that there is no sagging, and that the improvement in PCL dysfunction leads to better tension in the ACL (Fig 9). • Final fixation of the suture tape using the TensionLoc while the knee is in a flexed position at 90°.

ACL, anterior cruciate ligament; PCL, posterior cruciate ligament.

### Creation of 2 Posteromedial Portals

The procedure begins with the visualization of the posteromedial area from the anteromedial portal using a standard 30° arthroscope. Next, 2 Cathelin needles are inserted to create high and low posteromedial portals, with the low posteromedial portal created approximately 5 mm proximal to the medial meniscus and high posteromedial portal positioned as proximally to the medial meniscus as possible. Using the Cathelin needles as guides, an incision is made in the skin and joint capsule with a scalpel. A cannula is inserted into the low posteromedial portal. Finally, the arthroscope is introduced through the high posteromedial portal using a switching rod (Fig 3).

### Pull-Out Technique From Only Posteromedial Portals

The next step involves clearing the tibial bone tunnel creation area using a shaver or radiofrequency system from the low posteromedial portal. If there is significant displacement of the fracture, it is important to remove the debris from the fracture site. Subsequently, suture tape (ULTRATAPE; Smith & Nephew Endoscopy, Andover, MA) is sutured to proximal site of the PCL using a cinch stitch with a suture passer (FIRSTPASS MINI; Smith & Nephew Endoscopy) from the low posteromedial portal (Fig 4).

**Fig 6:**
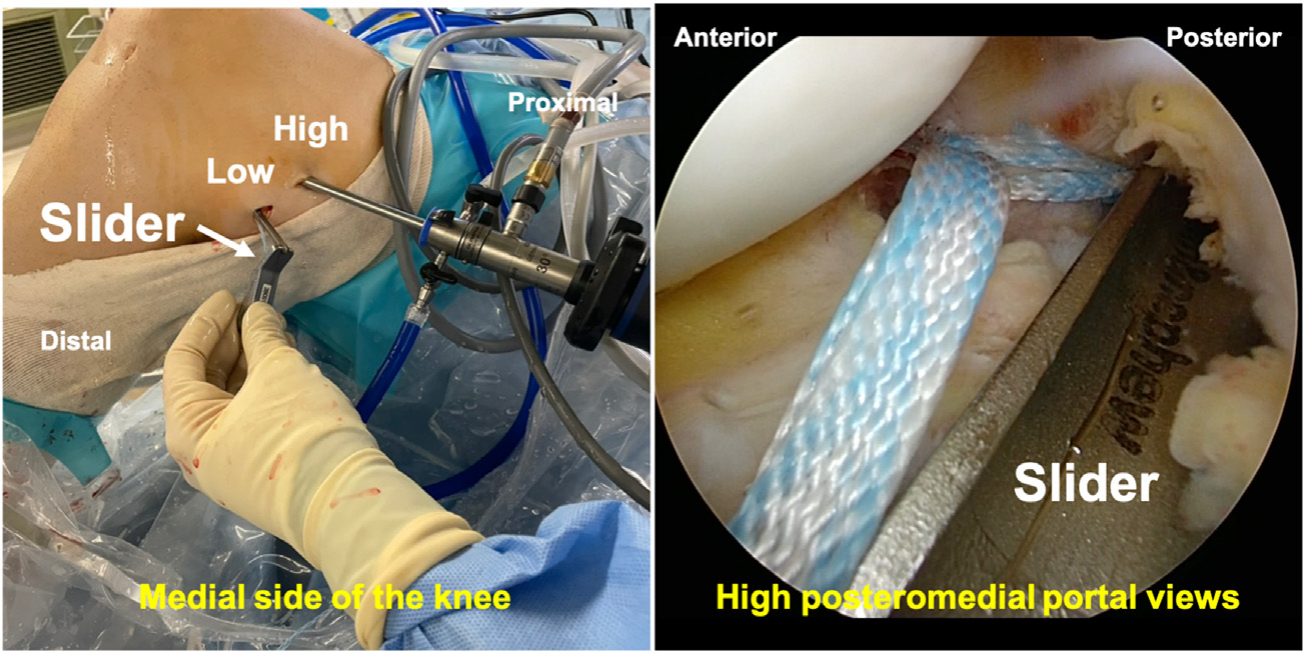
Using a slider for meniscal suturing instead of the cannula for the low posteromedial portal in the right knee.When the guide cannot pass through the cannula, the cannula in the low posteromedial portal is replaced with a slider for meniscal suturing, and the guide is inserted using this slider, positioned supine with the knee flexed at 90°, viewed from the high posteromedial portal.

**Fig 7:**
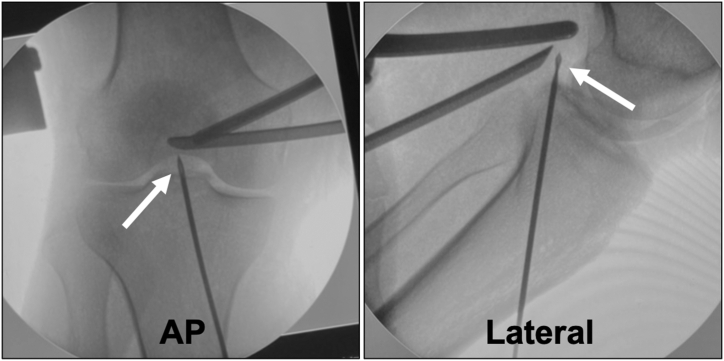
Confirming the position of the guide pin in the right knee using C-arm imaging.The position of the guide pin is confirmed using the anteroposterior (AP) and lateral views C-arm imaging (arrows).

A PCL guide is then inserted from the low posteromedial portal, and a guide pin (Smith & Nephew Endoscopy) is placed through a 2-cm skin incision on the medial side of the tibial tuberosity to the center of the main bone fragment (Fig 5). When the guide cannot pass through the cannula, the cannula in the low posteromedial portal is replaced with a slider for meniscal suturing, and the guide is inserted using this slider (Fig 6). If the PCL guide is bulky for the surgical space or has an insufficient angle, using an ACL femoral outside-in guide may be helpful. The position of the guide pin is confirmed using C-arm imaging (Fig 7), after which the tibial tunnel is created using an EndoButton drill (Smith & Nephew Endoscopy), indicating the guide pin.

**Fig 8:**
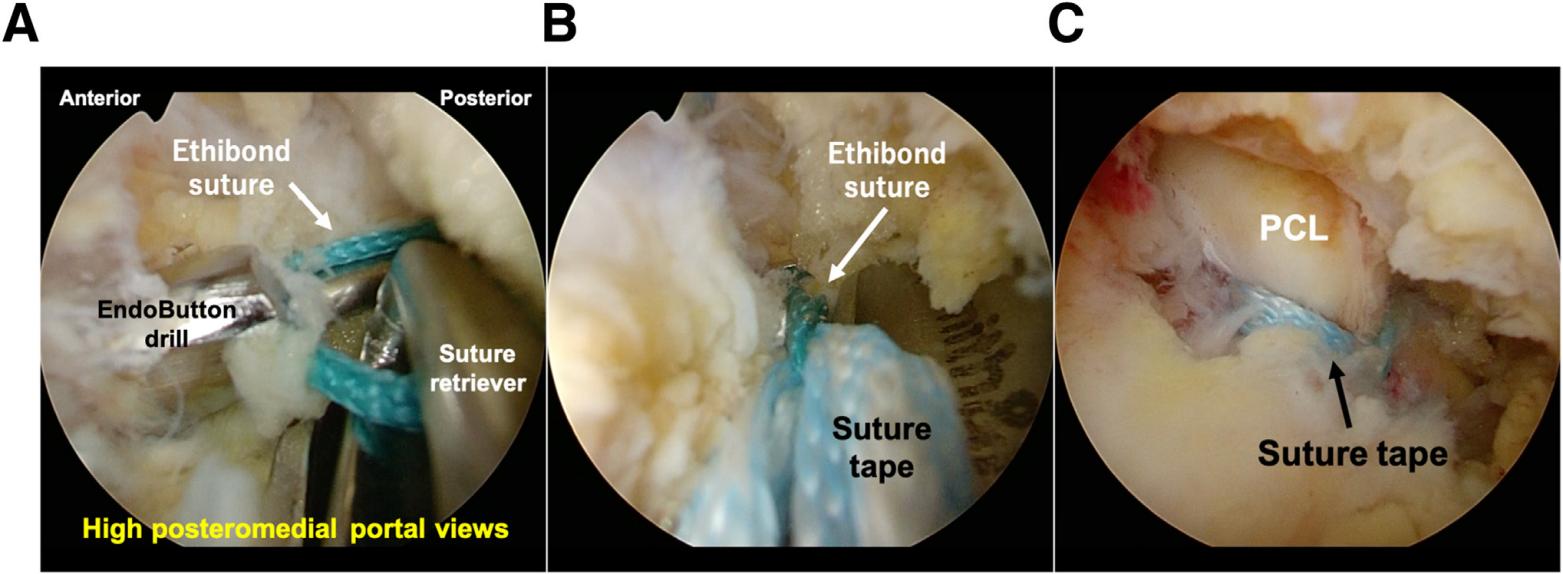
Pull-out of the suture tape in the right knee, positioned supine with the knee flexed at 90°, viewed from the high posteromedial portal. After creating the tibial tunnel with an EndoButton drill (Smith & Nephew Endoscopy) indicating the guide pin, the loop of the Ethibond suture (arrow) is passed through the anterior tibial bone tunnel using a suture passer and then retrieved through the low posteromedial portal with a suture retriever (A). The suture tape is relayed using a loop of Ethibond suture from the low posteromedial portal (B) to the tibial bone tunnel on the anterior surface of the tibia (C).

Next, the loop of No. 2 ETHIBOND suture is passed through the EndoButton drill from the anterior tibial surface using a suture retriever (Suture Retriever; Smith & Nephew Endoscopy) and then retrieved through the low posteromedial portal with a suture retriever (Loop Grasper; Smith & Nephew Endoscopy). The suture tape is relayed using a loop of ETHIBOND suture from the low posteromedial portal to the tibial bone tunnel on the anterior surface of the tibia (Fig 8).

### Tensioning and Fixation

**Fig 9:**
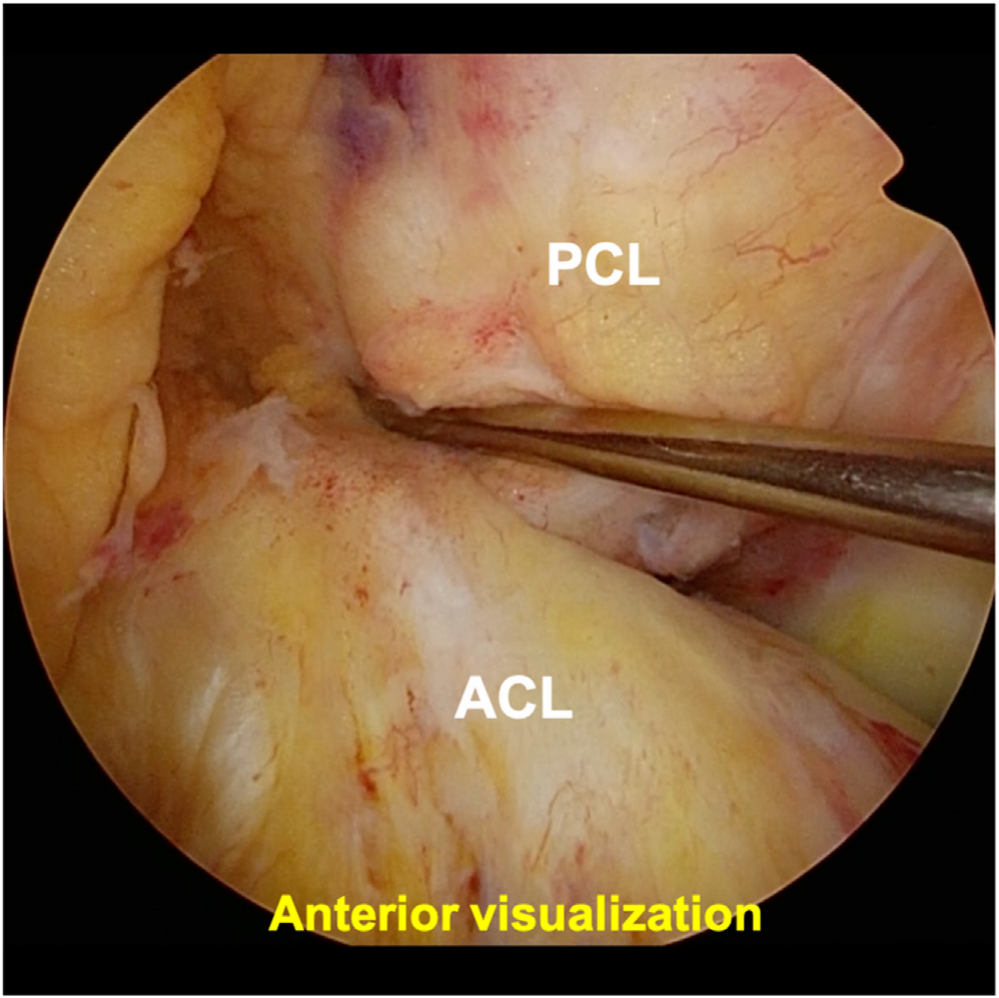
Tensioning and final fixation of the suture tape in the right knee, positioned supine with the knee flexed at 90°, viewed from the anterolateral portal. After temporary fixation of the suture tape using TensionLoc (Arthrex), tension is adjusted to the appropriate level. It is essential to ensure that the bone fragment does not float on C-arm imaging, that the posterior cruciate ligament (PCL) is properly tensioned under arthroscopic anterior visualization, that there is no sagging, and that improvement in PCL dysfunction leads to better tension in the anterior cruciate ligament (ACL). Neither the ACL nor the PCL is damaged compared to before surgery (Fig. 2). Once the appropriate tension is confirmed, final fixation of the suture tape is performed using TensionLoc while the knee is flexed at 90°.

In the tensioning and fixation phases, TensionLoc (Arthrex, Naples, FL) is installed in the anterior tibial bone tunnel, allowing temporary fixation of the suture tape. The tension is then adjusted to the appropriate level while confirming that the bone fragment is not floating through C-arm imaging. In addition, it is essential to confirm proper PCL tension under arthroscopic anterior visualization, and ensure that there is no sagging, and that improved PCL function leads to better ACL tension (Fig 9). Once the appropriate tension is confirmed, final fixation of the suture tape is performed using TensionLoc while the knee is flexed at 90°.

### Postoperative Management

**Fig 10:**
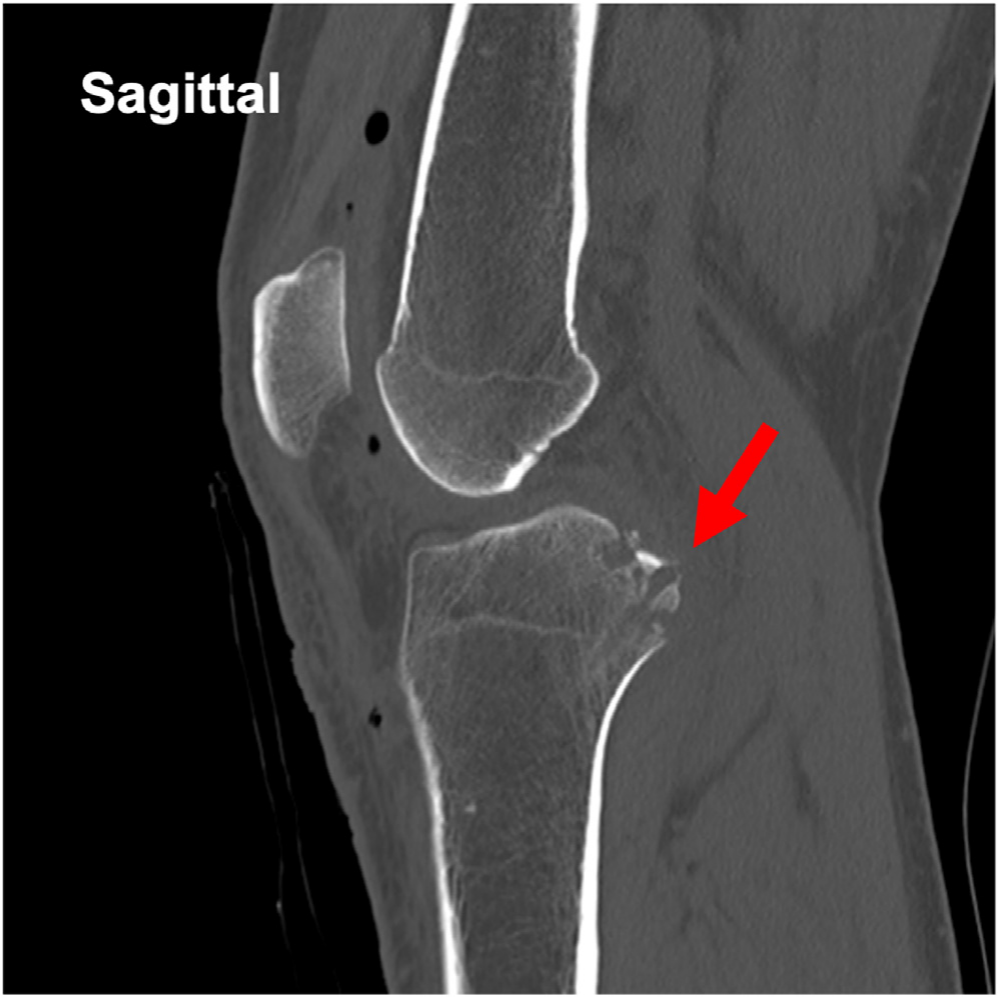
Postoperative sagittal computed tomography in the right knee.Anatomic reduction is obtained (arrow).

The knee is immobilized in full extension in a brace for 2 weeks after the operation, during which non−weight-bearing is implemented. From 2 weeks postoperatively, ROM training with a limit of less than 90° is initiated, and from 4 weeks postoperatively, ROM training without angular restrictions begins. Full weight-bearing is allowed 2 weeks postoperatively with a hinge brace. Postoperative CT shows anatomic reduction (Fig 10).

## Discussion

With the advancements in arthroscopic equipment, various arthroscopic techniques for treating PCL tibial avulsion fractures have been reported. These include methods that use complex suturing and suspensory fixation device.^8^, ^9^, ^10^, ^11^ Although various surgical fixation devices and techniques have been described for tibial eminence avulsion fractures, there is currently no established gold standard.

These techniques primarily use a combination of an anteromedial portal and a posteromedial portal. Therefore, it is necessary to pass devices, such as an arthroscopic camera and drill guide, through the space between the ACL and PCL to insert them into the PCL tibial attachment site, requiring clearance of the area between these ligaments. It is difficult to clear the synovium alone, and in several cases, there is a risk of damaging these ligaments. We herein proposed a method that minimizes invasion of ACL and PCL by creating 2 posteromedial portals and helps perform the entire pull-out technique through these 2 portals, thus eliminating the need to clear the space between ACL and PCL. Despite several reports on arthroscopic surgery using 2 posteromedial portals, all of these techniques involve the use of an anterior portal.^12^, ^13^, ^14^

There are some key points to this technique (Table 2). First, in this report, the PCL was sutured using a cinch stitch with a single artificial ligament pulled through a single tibial tunnel. However, this technique does not impose restrictions on the number of bone tunnels or sutures that may be used. By using 2 posteromedial portals in this technique, it was possible to perform various pull-out methods reported to date with minimal invasiveness, such as the crossover ties suturing technique, cross-linked pull-out suturing technique, EndoButton device (Smith & Nephew Endoscopy), and TightRope device (Arthrex).^8^, ^9^, ^10^, ^11^ Second, when the PCL guide is bulky or if the ACL tibial guide is inadequate in terms of angle, using an ACL femoral outside-in guide may yield better results. Finally, if instruments cannot pass through the cannula of the low posteromedial portal, using a slider for meniscal suturing may be beneficial.

**Table 2:** Key Points, Advantages, and Limitations of Procedures on the Bass of Experience

Key points • This technique does not impose restrictions on the number of bone tunnels or sutures that may be used. • By using 2 posteromedial portals in this technique, it is possible to perform various methods reported to date with minimal invasiveness. • If the PCL guide is bulky for the surgical space or has an insufficient angle, using an ACL femoral outside-in guide may be helpful. • If the instruments cannot pass through the cannula of the low posteromedial portal, using a slider for meniscus suturing can be beneficial (Fig 6).
Advantages • The technique is minimally invasive to the ACL and PCL. • The technique yields more adequate surgical field than conventional technique using anteromedial and posteromedial portals. • The technique is a safe and straightforward for the patient and does not require much time to perform.
Limitations • The threshold of suture tension for this procedure is unknown. • If there is considerable displacement of the bone fragment, there may be issues with surgical field space that could prevent the procedure from being performed.
Risks • As with other arthroscopic techniques that utilize posteromedial portals, there are risks of the popliteal neurovascular bundle injury and the development of arthrofibrosis.

ACL, anterior cruciate ligament; PCL, posterior cruciate ligament.

The advantages of this technique include that its minimally invasive impact ACL and PCL, more adequate surgical field than conventional technique using anteromedial and posteromedial portals, and its safety and straightforward application for the patient, which contributes to a relatively efficient procedure. However, this study has some limitations that must be acknowledged. The threshold for suture tension in this procedure remains undefined, and significant displacement of the bone fragment may pose challenges regarding the surgical field space, potentially complicating the execution of the procedure. However, even in this case, since the displacement of bone fragment is usually anterosuperior, the surgical field is likely to be better secured by this present technique than by conventional technique using anteromedial and posteromedial portals.

In this report, we described all the pull-out techniques performed through 2 posteromedial portals for PCL tibial avulsion fractures. This method is considered a useful surgical technique due to its minimal damage to the ACL and PCL, improved surgical field, and relative ease and safety of surgical technique.

## Disclosures

All authors (R.S., K.K., K.S., Y.H., T.N., T.H., M.N., H.M.) declare that they have no known competing financial interests or personal relationships that could have appeared to influence the work reported in this paper.

## Supplementary Data

Video 1 The currently used arthroscopic technique for PCL tibial avulsion fractur that uses anterior portals and a posteromedial portal is considered minimally invasive. However, it still requires clearing the space between the ACL and PCL to insert arthroscopic instruments, which can potentially damage these ligaments. One of the key points of this procedure is creating 2 posteromedial portals and performing the entire pull-out technique through these 2 portals, thus eliminating the need to clear the space between the ACL and PCL.The patient with a PCL tibial avulsion fractur in the right knee, under general anesthesia, is placed in the supine position on an operative table with a standard leg holder allowing full range of motion. First, standard knee arthroscopy is initiated with anterolateral and anteromedial portals. It is evident that the ACL is relaxed due to the effect of sagging. Next, two posteromedial portals are created with the knee flexed at 90°. The posteromedial area is visualized from the anteromedial portal, and two Cathelin needles are inserted to establish high and low posteromedial portals. The low portal is positioned approximately 5 mm proximal to the medial meniscus, while the high portal is as proximal as possible. The skin and joint capsule are incised with a scalpel, and a cannula is inserted into the low posteromedial portal. The arthroscope is then introduced through the high posteromedial portal using a switching rod.In a 90° flexed knee position, visualize the PCL from the high posteromedial portal. For the pull-out technique, the tibial bone tunnel area is cleared using a shaver or radio frequency system from the low posteromedial portal. If fracture displacement is significant, debris from the fracture site is also removed. The suture tape is sutured to the PCL using suture passer from the low posteromedial portal. In this case, we used a Chinch stitch suturing. A PCL guide is inserted from the low portal, and a guide pin is placed from the medial side of the tibial tuberosity. If the guide cannot pass through the cannula, it is replaced with a slider for meniscus suturing. The position of the guide pin is confirmed with C-arm imaging, followed by creating the tibial tunnel with a 4.5-mm cannulated drill. The loop of the Ethibond is passed through the anterior tibial bone tunnel using a suture passer and retrieved through the low posteromedial portal. The suture tape is then relayed using this loop.During the tensioning and fixation phase, the TensionLoc is installed in the anterior tibial bone tunnel for temporary fixation of the suture tape in a 90° flexed knee position. Tension is adjusted to the appropriate level, ensuring the bone fragment is stable and confirming proper tension on the PCL under arthroscopic visualization. Once confirmed, final fixation of the suture tape is achieved. Finally, during the anterior arthroscopic visualization from the anterolateral portal, it is confirmed that sagging is resolved and the ACL is properly tensioned, while also ensuring that both the ACL and PCL are completely preserved.
